# Post‐remission cytopenia management in patients with AML treated with venetoclax in combination with hypomethylating agents: Pre‐ versus post‐VIALE‐A real‐world experience from a predominantly US community setting

**DOI:** 10.1002/cam4.6430

**Published:** 2023-08-11

**Authors:** Pankit Vachhani, Esprit Ma, Tao Xu, Melissa Montez, Sarah Worth, Archibong Yellow‐Duke, Wei‐Han Cheng, Michael E. Werner, Jonathan Abbas, William Donnellan

**Affiliations:** ^1^ O'Neal Comprehensive Cancer Center at the University of Alabama at Birmingham Birmingham Alabama USA; ^2^ Genentech, Inc. South San Francisco California USA; ^3^ F. Hoffmann‐La Roche Ltd Basel Switzerland; ^4^ AbbVie, Inc. North Chicago Illinois USA; ^5^ Tennessee Oncology Nashville Tennessee USA

**Keywords:** acute myeloid leukemia, bone marrow assessment, hypomethylating agents, real‐world, schedule modifications, survival outcomes, treatment management, venetoclax

## Abstract

**Background:**

This retrospective cohort study used an electronic health record‐derived, de‐identified, US patient‐level database to better understand the real‐world treatment experience, in a predominantly community setting (80.3% of patients), of venetoclax+hypomethylating agents (HMAs) in routine clinical care, pre‐ and post‐VIALE‐A, to determine whether the post‐remission cytopenia management insight from VIALE‐A was reflected in real‐world clinical practice.

**Methods:**

Patients with newly diagnosed acute myeloid leukemia (AML; *N* = 498), who initiated venetoclax+HMA ≤30 days from AML diagnosis from June 1, 2018, to March 31, 2021, were stratified into pre‐(*n* = 330) and post‐(*n* = 168) VIALE‐A cohorts.

**Results:**

More patients in the post‐(61%) versus pre‐(45%) VIALE‐A cohort had their first biopsy by 28 ± 14 days post‐treatment initiation. Patients underwent bone marrow (BM) assessment earlier in the post‐ versus pre‐VIALE‐A cohort, and first identification of response was also earlier (2.5 vs 5.1 months, respectively). More venetoclax schedule modifications post‐remission occurred among post‐(82.1%) versus pre‐(73.8%) VIALE‐A responders; the most common reason for modification was treatment toxicities, specifically cytopenia. Treatment survival outcomes were comparable with or without venetoclax schedule modifications.

**Conclusions:**

Findings suggest that venetoclax schedule modifications can be used to manage cytopenia events without adversely affecting outcomes. Opportunities remain to improve earlier BM assessment to determine venetoclax schedule modifications, providing the best chance for optimal treatment outcomes.

## Graphical abstract

This retrospective study assesses whether the post‐remission cytopenia management insight from VIALE‐A was reflected in real‐world clinical practice. Findings are consistent with VIALE‐A: Venetoclax schedule modifications can manage cytopenia events without adversely affecting outcomes.

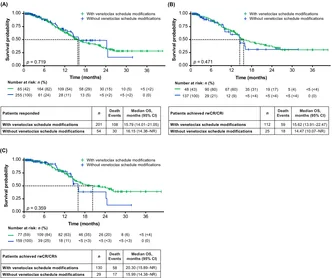

## 1 INTRODUCTION

Acute myeloid leukemia (AML) is an aggressive and life‐threatening hematologic malignancy. In 2018, venetoclax in combination with hypomethylating agents (HMAs), such as azacitidine (Aza) and decitabine, received accelerated approval for the treatment of patients with newly diagnosed AML who are ≥75 years of age or ineligible for intensive chemotherapy based on results from the phase 1b (NCT02203773) study.^1^ Full approval followed in 2020, based on the results from the phase 3 (VIALE‐A; NCT02993523) study,^2^ which evaluated venetoclax in combination with Aza versus placebo+Aza.

The VIALE‐A study demonstrated longer median overall survival (OS; 14.7 months vs 9.6 months; hazard ratio [HR]: 0.7, 95% confidence interval [CI]: 0.5–0.9, *p* < 0.001), higher composite complete remission rates (66.4% [95% CI: 60.6–71.9] vs 28.3% [95% CI: 21.1–36.3], *p* < 0.001), and shorter median time to first response (1.3 months [range: 0.6–9.9] vs 2.8 months [range: 0.8–13.2]) for patients treated with venetoclax+Aza versus placebo+Aza, respectively.^2^ Higher rates of hematologic adverse events (AEs; including thrombocytopenia, neutropenia, and anemia; 83.4% vs 69.4%, respectively) were also observed for patients treated with venetoclax+Aza versus placebo+Aza.^2^ Results from the VIALE‐A post‐remission cytopenia management analysis highlight the importance of early assessment of response to implement appropriate venetoclax+Aza dosing schedule modifications, delays, and interruptions to manage cytopenia without negatively impacting treatment outcomes.^3^, ^4^

While early real‐world experience with venetoclax+HMAs has been evaluated,^5^, ^6^, ^7^, ^8^, ^9^ there are limited data on real‐world experience of venetoclax following the results of the phase 3 VIALE‐A trial. This study aims to better understand real‐world post‐remission cytopenia management experience of venetoclax+HMAs in a predominantly community care setting, pre‐ and post‐VIALE‐A.

## 2 METHODS

### 2.1 Data source

This retrospective cohort study used data from the Flatiron Health database: a US nationwide, electronic health record (EHR)‐derived longitudinal database, which comprised of de‐identified patient‐level data from approximately 280 US cancer clinics (~800 sites of care) curated via technology‐enabled abstraction.^10^, ^11^ Practice type was either community or academic, so community‐based clinics that were academic‐affiliated were categorized as academic. The Flatiron Health AML database includes patients diagnosed with AML (ICD 10: C92.0x, C92.4x, C92.5x, C92.6x, C92.9x, C92.Ax, C93.0x, C94.0x, C94.2x, C94.4x) who had at least two documented clinical visits for AML (academic or community encounters on different days). Institutional review board approval of the study protocol was obtained prior to study conduct and included a waiver of informed consent. Studies were conducted in accordance with recognized ethical guidelines including the Declaration of Helsinki, the Council for International Organizations of Medical Science (CIOMS), the Belmont Report, and the US Common Rule.

### 2.2 Patient cohort

Patients included in this study were selected from the Flatiron Health database. Patients were included if they were aged ≥18 years, newly diagnosed with AML, and initiated venetoclax+HMA treatment ≤30 days from AML diagnosis between June 1, 2018, and March 31, 2021. Exclusion criteria included patients who were diagnosed with acute promyelocytic leukemia and patients with a positive t(9;22) cytogenetic test on or before start date of first‐line (1L) AML treatment. Further, patients who received venetoclax more than 14 days prior to AML diagnosis, received HMAs before AML diagnosis, or received a 1L regimen containing a clinical study drug were also excluded from the study.

Venetoclax+HMA treatment initiation was used as the study index date (Data S1). Patients who met the study criteria were stratified into two groups with respect to when the phase 3 VIALE‐A results were presented at the European Hematology Association Congress in June 2020^12^: (i) the pre‐VIALE‐A cohort, which initiated 1L venetoclax+HMA between June 1, 2018, and June 30, 2020; and (ii) the post‐VIALE‐A cohort, which initiated 1L venetoclax+HMA between July 1, 2020, and March 31, 2021. This stratification was used to determine whether the experience on treatment management derived from the VIALE‐A trial (the importance of early BM assessment and the use of dosing schedule modifications) was reflected in clinical practice. Additionally, subgroup analyses were conducted among patients whose treatment dose schedule per cycle was modified by converting from 28 days to 21 days (21/28) in the next treatment cycle after remission versus patients who remained on 28‐day cycles after remission.

### 2.3 Variables

European LeukemiaNet (ELN) 2017 risk classification^13^ was derived from available cytogenetic and molecular data in the database. Timing of bone marrow (BM) biopsy, response (<5% BM blasts), real‐world complete response/complete response with partial hematologic recovery (rwCR/CRh; <5% BM blasts with a platelet count >50 × 10^9^/L and absolute neutrophil count [ANC] >0.5 × 10^9^/L), and rwCR/complete response with incomplete hematologic recovery (rwCR/CRi; <5% BM blasts with either platelet count <100 × 10^9^/L or ANC <1000 × 10^9^/L) were measured. Treatment duration was quantified in terms of time to last administration (TTLA), defined as time from 1L treatment initiation to last administration before an absence of treatment (event date or censor date) or death (discontinuation event). Additional information related to venetoclax treatment experience was manually abstracted from unstructured data; this included reasons for and patterns of treatment schedule modifications (e.g., any in‐cycle interruptions, cycle delays, or dose schedule per cycle changes, Data S1). Further detailed information on the days of treatment dose schedule per cycle for venetoclax was abstracted from physician notes.

### 2.4 Statistical analysis

Descriptive statistics were calculated to assess patient characteristics and demographics, and treatment schedule modifications after achieving remission were examined. Kaplan–Meier analyses summarized TTLA, time to first BM biopsy, time to first response (<5% BM blasts), and time from start of venetoclax treatment to end of treatment, death, or censoring at the end of follow‐up at data cutoff on January 31, 2022, whichever came first. Cox regression time‐varying analyses were conducted to examine the association between venetoclax treatment schedule modifications after remission and OS. Log‐rank test was used to compare time to event endpoints between pre‐ versus post‐ VIALE‐A cohorts and between patients with versus without treatment schedule modifications. All statistical analyses were performed using the R language and software environment, version 3.6.3.

## 3 RESULTS

In total, 498 patients treated with venetoclax+HMAs met the study criteria (Data S1). Overall, 66.3% (330/498) of patients were pre‐VIALE‐A and 33.7% (168/498) were post‐VIALE‐A. Similar baseline patient characteristics were reported for these two cohorts (Table 1); for pre‐ versus post‐VIALE‐A, respectively, median age was 76 versus 75 years and secondary AML was 33.3% versus 27.4%. Most patients were treated with 1‐L venetoclax+HMAs in community practice (80.3% of patients in the pre‐VIALE‐A cohort and 80.4% of patients in the post‐VIALE‐A cohort) versus academic settings. ELN 2017 classifications for the pre‐ versus post‐VIALE‐A cohorts, respectively, were 41.8% versus 45.8% for adverse, 21.5% versus 28.0% for intermediate, and 13.0% versus 8.9% for favorable.

**TABLE 1:** Patient baseline demographics and characteristics by pre‐ versus post‐VIALE‐A.

	Pre‐VIALE‐A (*n* = 330)	Post‐VIALE‐A (*n* = 168)	*p* Value
Age at diagnosis			
Mean (SD)	74.9 (7.1)	73.8 (7.8)	0.12
Median (min, max)	76.0 (39.0, 83.0)	75.0 (36.0, 84.0)	
Age at diagnosis (categorical), *n* (%)			
<18 years	0	0	0.189
18–64 years	28 (8.5)	17 (10.1)	
65–74 years	103 (31.2)	64 (38.1)	
≥75 years	199 (60.3)	87 (51.8)	
Sex, *n* (%)			
Female	133 (40.3)	71 (42.3)	0.746
Male	197 (59.7)	97 (57.7)	
Diagnosis year, *n* (%)			
2018	39 (11.8)	0	<0.001^a^
2019	183 (55.5)	0	
2020	108 (32.7)	114 (67.9)	
2021	0	54 (32.1)	
AML type, *n* (%)			
De novo AML	220 (66.7)	122 (72.6)	0.211
Secondary AML	110 (33.3)	46 (27.4)	
Practice type, *n* (%)			
Academic	65 (19.7)	33 (19.6)	1
Community	265 (80.3)	135 (80.4)	
Prior malignancy, *n* (%)			
Yes	230 (69.7)	109 (64.9)	0.323
No	100 (30.3)	59 (35.1)	
Prior malignancy diagnosis, *n* (%)			
CMML/CMMoL	10 (3.0)	<5 (<3.0)^b^	0.61
ET	5 (1.5)	<5 (<3.0)^b^	
MDS	85 (25.8)	37 (22.0)	
MF	<5 (<1.5)^b^	<5 (<3.0)^b^	
Other	<5 (<1.5)^b^	<5 (<3.0)^b^	
PV/PVC	<5 (<1.5)^b^	<5 (<3.0)^b^	
Unknown/not documented	0	<5 (<3.0)^b^	
Missing	220 (66.7)	122 (72.6)	
Has blast count data for diagnosis, *n* (%)			
Yes	315 (95.5)	165 (98.2)	0.191
No	15 (4.5)	<5 (<3.0)^b^	
Bone marrow blast at diagnosis, *n* (%)			
<5%	1 (0.3)	<5 (<3.0)^b^	NA
6–20%	37 (11.2)	25 (14.9)	
21–30%	76 (23.0)	44 (26.2)	
31–50%	77 (23.3)	37 (33.9)	
>50%	124 (37.6)	57 (33.9)	
Unknown/not documented	15 (4.5)	<5 (<3.0)^b^	
ECOG PS, *n* (%)			
0	70 (21.2)	34 (20.2)	0.73
1	133 (40.3)	80 (47.6)	
2	59 (17.9)	30 (17.9)	
3+	15 (4.5)	6 (3.6)	
Missing	53 (16.1)	18 (10.7)	
ELN classification, *n* (%)			
Adverse	138 (41.8)	77 (45.8)	0.183
Intermediate	71 (21.5)	47 (28.0)	
Favorable	43 (13.0)	15 (8.9)	
Inconclusive	49 (14.8)	17 (10.1)	
Missing	29 (8.8)	12 (7.1)	
Transplant after treatment initiation, *n* (%)			
Yes	24 (7.3)	13 (7.7)	0.0705
Missing^c^	306 (92.7)	155 (92.3)	
Somatic mutations, *n* (%)			
*FLT3*			
Negative	166 (50.3)	92 (54.8)	0.248
Positive	37 (11.2)	13 (7.7)	
Missing^c^	127 (38.5)	63 (37.5)	
*IDH1*			
Negative	135 (40.9)	79 (47.0)	0.513
Positive	22 (6.7)	9 (5.4)	
Missing^c^	173 (52.4)	80 (47.6)	
*IDH2*			
Negative	114 (34.5)	66 (39.3)	0.331
Positive	55 (16.7)	23 (13.7)	
Missing^c^	161 (48.8)	79 (47.0)	
*NPM1*			
Negative	141 (42.7)	74 (44.0)	0.247
Positive	27 (8.2)	8 (4.8)	
Missing^c^	162 (49.1)	86 (51.2)	
*TP53*			
Negative	76 (23.0)	45 (26.8)	0.469
Positive	33 (10.0)	26 (15.5)	
Missing^c^	221 (67.0)	97 (57.7)	

Abbreviations: AML, acute myeloid leukemia; CMML/CMMoL, chronic myelomonocytic leukemia; ECOG PS, Eastern Cooperative Oncology Group performance status; ELN, European LeukemiaNet; ET, essential thrombocytopenia; *FLT3*, Fms Related Receptor Tyrosine Kinase 3; *IDH*, isocitrate dehydrogenase; Max, maximum; MDS, myelodysplastic syndrome; MF, myelofibrosis; Min, minimum; *NPM1*, Nucleophosmin 1; PV/PVC, polycythemia vera; SD, standard deviation; *TP53*, tumor protein 53.

^a^ *p* Value expected as the population was divided based on a time factor, thus not much value should be drawn from this.

^b^ Per the threshold accepted by the National Center for Health Statistics and the Agency for Healthcare Research and Quality, detailed in the Federal Committee's Statistical Policy, 2005, any instances where there are fewer than five patients for a particular characteristic or variable have been described as such to eliminate potential patient reidentification.

^c^ Missing includes unknown or not documented in the database.

The median time from diagnosis to venetoclax treatment initiation was 12 days for the pre‐VIALE‐A cohort and 14 days for the post‐VIALE‐A cohort. Median TTLA was 4.3 months (median follow‐up: 9.0 months) and 4.7 months (median follow‐up: 8.5 months), for pre‐ versus post‐VIALE‐A, respectively. Fewer patients in the post‐ versus pre‐VIALE‐A cohort (13.1% [22/168] vs 19.1% [63/330], respectively) discontinued ≤60 days after treatment initiation (*p* = 0.10). Among patients with documented reasons for venetoclax treatment discontinuation in physician notes, the most common cited reasons included treatment toxicities, other reasons (e.g., non‐cancer‐related medical issues and cancer‐related treatment), and progression (Table 2).

**TABLE 2:** Reasons for treatment discontinuation by pre‐ versus post‐VIALE‐A.

	Pre‐VIALE‐A (*n* = 99)	Post‐VIALE‐A (*n* = 42)
Toxic effect of treatment, *n* (%)	49 (49.5)	15 (35.7)
Neutropenia	42 (42.4)	10 (23.8)
Thrombocytopenia	28 (28.3)	10 (23.8)
Anemia	25 (25.3)	6 (14.3)
Other	7 (0.7%)	<5 (<12)^b^
Other reasons,^a^ *n* (%)	24 (24.2)	15 (35.7)
Progression, *n* (%)	23 (23.2)	11 (25.2)
Completed treatment, *n* (%)	6 (6.1)	<5 (<12)^b^
Disease‐related symptoms not related to treatment, *n* (%)	5 (5.1)	<5 (<12)^b^

^a^ Including but not limited to non‐cancer‐related medical issues (e.g., patients hospitalized for non‐drug related issues) and cancer‐related treatments (e.g., stopping treatment prior to transplant procedure).

^b^ Per the threshold accepted by the National Center for Health Statistics and the Agency for Healthcare Research and Quality, detailed in the Federal Committee's Statistical Policy, 2005, any instances where there are fewer than five patients for a particular characteristic or variable have been described as such to eliminate potential patient reidentification.

**FIGURE 1:**
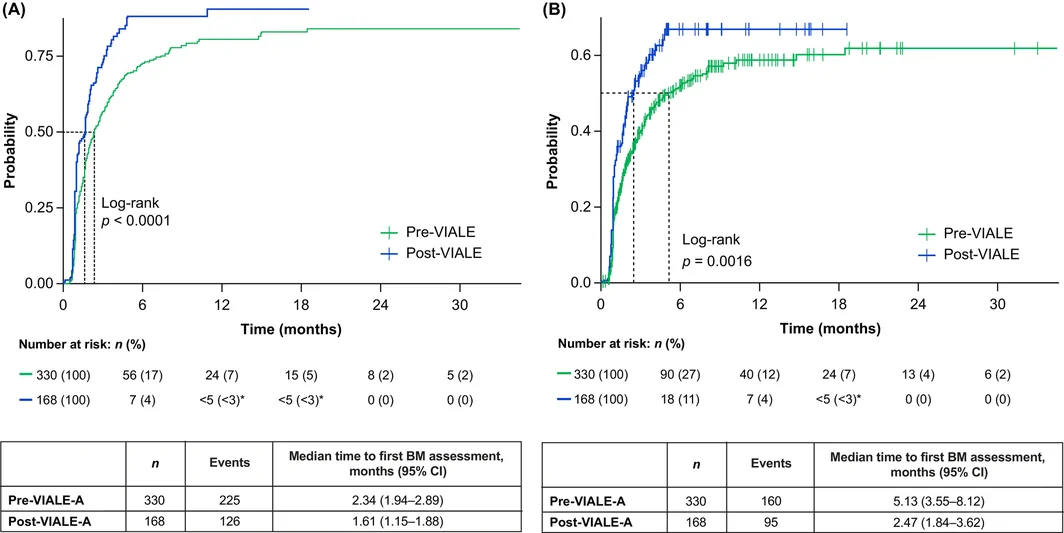
Time to first BM assessment (A) and first response (B) for the pre‐ versus post‐VIALE‐A cohorts. BM, bone marrow; CI, confidence interval.

More patients in the post‐ versus pre‐VIALE‐A cohort (61.1% [77/126] vs 44.9% [101/225]) had their first BM biopsy by 28 ± 14 days after treatment initiation (proxy for treatment cycle 1); all except one patient achieved response by first BM assessment. Patients in the post‐VIALE‐A cohort underwent BM assessment earlier than patients in the pre‐VIALE‐A cohort (median time 1.6 months vs 2.8 months, respectively; Figure 1A). The first identification of response was also earlier in patients in the post‐ versus pre‐VIALE‐A cohort (2.5 months vs 5.1 months, respectively, *p* < 0.0001; Figure 1B). More patients in the post‐ versus the pre‐VIALE‐A cohort had venetoclax schedule modification post‐remission (82.1% vs 73.8%, respectively; Data S1). The most common reasons (reported in >20.0% of patients) for venetoclax schedule modifications after remission, as documented in available physician notes (122 pre‐ vs 79 post‐VIALE‐A), were treatment toxicities and other reasons (e.g., non‐cancer‐related medical issues and cancer‐related treatment; Table 3).

**TABLE 3:** Reasons for treatment modifications after remission by pre‐ versus post‐VIALE‐A.

	Pre‐VIALE‐A (*n* = 122)	Post‐VIALE‐A (*n* = 79)
Toxic effect of treatment, *n* (%)	107 (87.7)	71 (89.9)
Neutropenia	98/107 (91.6)	67/71 (94.4)
Thrombocytopenia	46/107 (43.0)	38/71 (53.5)
Anemia	46/107 (43.0)	38/71 (53.5)
Other	17/107 (15.9)	11/71 (15.5)
Other reasons,^a^ *n* (%)	60 (49.2)	40 (50.6)
Disease‐related symptoms not due to therapy, *n* (%)	6 (4.9)	<5 (<6)^b^
Drug–drug interactions, *n* (%)	6 (4.9)	<5 (<6)^b^

^a^ Including but not limited to non‐cancer‐related medical issues (e.g., patients hospitalized for non‐drug related issues) and cancer‐related treatments (e.g., stopping treatment prior to transplant procedure).

^b^ Per the threshold accepted by the National Center for Health Statistics and the Agency for Healthcare Research and Quality, detailed in the Federal Committee's Statistical Policy, 2005, any instances where there are fewer than five patients for a particular characteristic or variable have been described as such to eliminate potential patient reidentification.

**FIGURE 2:**
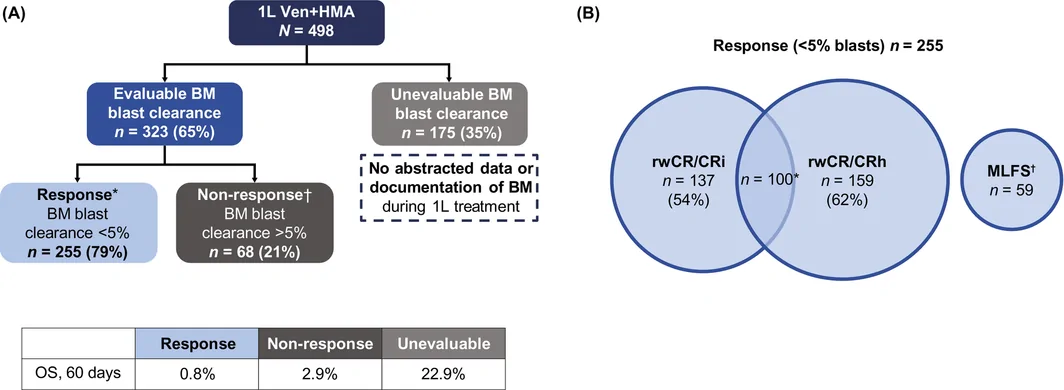
Patients with newly diagnosed AML, who initiated venetoclax+HMA as 1L treatment ≤30 days from AML diagnosis, by BM blast clearance (A) and proportion of patients who achieved remission (B). *100 patients achieved rwCR/CRi and rwCR/CRh. ^†^ < 5% blasts, absence of blasts with Auer rods, absence of extramedullary disease, no hematologic recovery required. 1L, first‐line; AML, acute myeloid leukemia; BM, bone marrow; CRh, complete response with partial hematological recovery; CRi, complete response with incomplete hematological recovery; HMA, hypomethylating agent; MLFS, morphologic leukemia‐free state; rwCR, real‐world complete response.

Overall, 64.9% (323/498) of patients with evaluable BM blast clearance were documented during follow‐up; these patients had low early mortality (death ≤60 days; 1.2% [4/323]). The remaining 35.1% (175/498) without an evaluable BM blast clearance (no abstracted data or documented BM during 1L treatment) had higher early mortality (60‐day mortality: 22.9% [40/175]), a probable reason for no BM documentation. Responses (blast <5%) were reported for 78.9% (255/323) of patients with evaluable BM assessment (Figure 2A). Of these, 53.7% (137/255) achieved rwCR/CRi and 62.4% (159/255) achieved rwCR/CRh (Figure 2B). Notably, 100 patients met the criteria for both rwCR/CRi and rwCR/CRh (Figure 2B).

**FIGURE 3:**
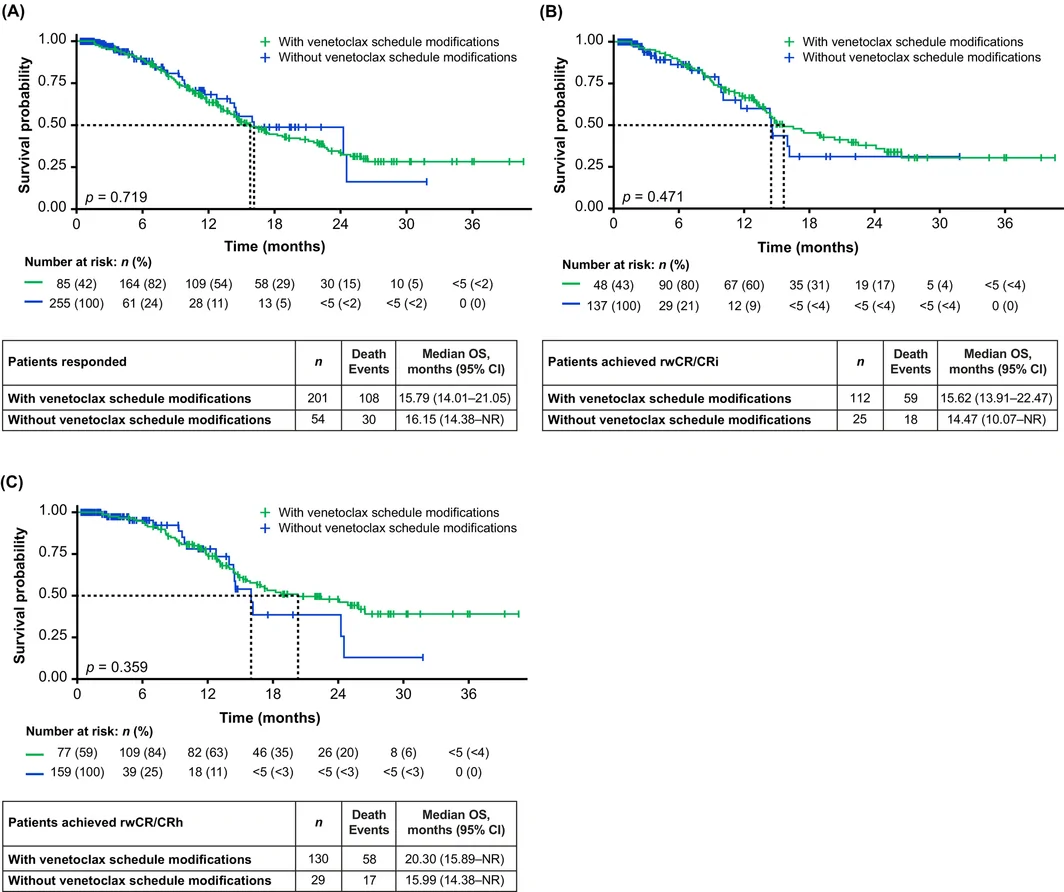
Time‐varying survival analysis of patients with newly diagnosed AML receiving venetoclax+HMA, with or without treatment schedule modifications after remission for all patients who responded (A), patients who achieved rwCR/CRi (B) and patients who achieved rwCR/CRh (C). 100 patients achieved rwCR/CRi and rwCR/CRh. AML, acute myeloid leukemia; CI, confidence interval; CRh, complete response with partial hematological recovery; CRi, complete response with incomplete hematological recovery; HMA, hypomethylating agent; NR, not reached; OS, overall survival; rwCR, real‐world complete response.

Patients with or without venetoclax schedule modification after remission had similar patient characteristics (Data S1). The time‐varying survival analysis showed that treatment outcomes were comparable for patients with or without venetoclax treatment schedule modifications among patients who achieved response or rwCR/CRi or rwCR/CRh (Figure 3).

In total, 79.3% (395/498) of patients had data for venetoclax treatment initiation specifying the use of a 28‐day schedule for cycle 1, among which 184 patients achieved response. Of these patients, 73.4% (135/184) stayed on 28‐day dosing cycles after achieving response, while 10.9% (20/184) of patients had their venetoclax treatment schedule converted from a 28‐day to a 21‐day dosing duration post‐remission (10 patients who achieved rwCR/CRi and 14 patients who achieved rwCR/CRh; Data S1). The death events were low, where some median OS and upper bound 95% CI were not reached in the time‐varying survival analysis at the time of the analysis (Data S1). Notably, 15.8% (29/184) of patients changed to other schedules (14‐day dosing cycles).

## 4 DISCUSSION

The results from the VIALE‐A trial have led to more patients being treated with venetoclax+Aza and a better understanding of patient management principles, including early BM assessment. This study aimed to assess whether this has translated into better clinical practice in real‐world settings over time by comparing data pre‐ and post‐VIALE‐A. This study observed an evolution of earlier BM biopsy assessments, fewer patients with early discontinuation (≤60 days after treatment initiation), and an improvement in management of venetoclax treatment by implementation of appropriate schedule modification in patients with newly diagnosed AML who received venetoclax+HMAs from pre‐ to post‐VIALE‐A, in a predominantly community setting in the US.

Treatment toxicities, in particular cytopenias, was cited as a common reason for venetoclax treatment schedule modification post‐remission, and the similar treatment outcomes observed in patients with or without venetoclax treatment schedule modification after remission in this study are consistent with the findings from the VIALE‐A post‐remission cytopenia management analysis.^3^, ^4^ It should be noted that this real‐world cohort is more diverse compared with patients enrolled in the VIALE‐A trial,^2^ as it includes more patients with secondary AML (31% vs 25%, respectively) and more patients with prior malignancies (30% vs unspecified numbers in VIALE‐A as patients with malignancies within 2 years prior to study entry [with a few exceptions] are excluded). The overall 30‐day mortality was low (2% in this study vs 7% for venetoclax+Aza in the VIALE‐A trial). Further, a higher proportion of the venetoclax+HMA‐treated patients in this real‐world cohort underwent transplant (7%; 37/498) compared with VIALE‐A (<1%; 2/283). The differences in the routine clinical care population versus patients enrolled in VIALE‐A are not surprising given that clinical trials have selective eligibility criteria and tightly controlled settings.^14^

Despite variations in routine clinical practices, findings from this study align with several publications on early experience of venetoclax in AML in the pre‐VIALE‐A era, which emphasize the importance of appropriate venetoclax management for cytopenias to optimize patient outcomes.^6^, ^7^, ^8^, ^9^ Data from our recent publication on the early experience of venetoclax during the time frame from June 2018 to the end of January 2020 highlighted the importance of treatment schedule modifications to optimize patient outcomes in a predominately community setting.^5^ The AML real‐world evidence (ARC) initiative from academic centers (10 sites based in the US and four sites in Israel) also highlighted that a delay in timing of BM assessments may affect the evaluation of time to response and post‐response management in the real‐world.^9^ While there are several variations in routine clinical practice including converting venetoclax dosing duration from 28 days to 21 or 14 days, adding growth factor (granulocyte colony‐stimulating factor [G‐CSF]), incorporating interruptions between cycles (with or without growth factor support) and reducing the HMA dose (often by half),^6^, ^7^, ^8^, ^9^ earlier BM assessment is the common shared perspective in managing cytopenias post‐remission. It is therefore encouraging to observe in this study, in a predominately community setting, an increase in the proportion of patients with early BM biopsy (28 ± 14 days after initiation) compared with the earlier experience. However, the 61.1% of patients in the post‐VIALE‐A cohort with early BM biopsy demonstrate opportunities remain to ensure that all patients have early BM assessment, to inform treatment schedule modifications post‐remission to manage cytopenias.

Studies to date suggest that utilization of dose schedule modifications for venetoclax is important for both short‐term and long‐term patient outcomes. In the post hoc VIALE‐A post‐remission cytopenia analysis, responders who converted to 21/28‐day cycles early (in the next cycle post‐remission) had fewer transfusions compared with patients who converted to 21/28‐day cycles later (in subsequent cycles). Further, overall the conversion to 21/28‐day dosing of venetoclax did not adversely impact outcomes.^4^ In this study, only a minor subset (<15%) of patients converted from 28‐ to 21‐day dosing cycles post‐remission and death events were low, so some median OS and upper bound 95% CI were not reached at the time of the analysis. Future studies are warranted to evaluate the association of early conversion post‐remission to 21/28‐day venetoclax cycles on treatment outcomes in routine clinical care.

There are several limitations of this study. The Flatiron Health (EHR‐derived) database used was not specifically designed to evaluate the research questions framed. Thus, a lack of documentation of treatment or procedures does not mean that these did not occur (e.g., BM assessments, documentation of reasons for treatment schedule modifications, treatment cycle, treatment dose schedule per cycle for HMA, etc.), but rather that they were not logged in the patient medical records. Further, due to the open cohort nature of the real‐world database, data on patients' treatment outside of the Flatiron Health network (at other sites of care) may not be captured and therefore may lead to underreporting or missing data. There were limited data on concomitant medication (e.g., G‐CSF, anti‐infective medications to prevent recurrent viral, bacterial, or fungal infections, and Cytochrome P450 3A4 inhibitors) to evaluate potential drug–drug interactions. With the lack of baseline comorbidity, we were not able to quantify these real‐world patients' eligibility for intensive chemotherapy.^15^ Findings are reflective of practices within the Flatiron Health network only and may be distinctly different from other centers, thus limiting the generalizability of the study. In addition, while the Flatiron Health network is predominately community based and included some group practices, not all commsites unanimously practice the same; thus, our study findings are subject to practice variations.

## 5 CONCLUSION

Findings from this study are consistent with those from the VIALE‐A post‐remission cytopenia management analysis. Early BM biopsy is critical to assess response in order to inform the implementation of venetoclax treatment schedule modifications. The comparable time‐varying survival observed in this study demonstrates the effectiveness of venetoclax treatment schedule modifications to manage cytopenia events without adversely affecting outcomes. Opportunities remain to further increase the number of patients with early BM assessment to determine whether venetoclax treatment schedule modifications are necessary to provide the best chance for optimal treatment outcomes for patients.

## AUTHOR CONTRIBUTIONS

**Pankit Vachhani:** Conceptualization (equal); formal analysis (equal); supervision (equal); writing – review and editing (equal). **Esprit Ma:** Conceptualization (equal); formal analysis (equal); funding acquisition (equal); supervision (equal); writing – review and editing (equal). **Tao Xu:** Conceptualization (equal); formal analysis (equal); writing – review and editing (equal). **Melissa Montez:** Conceptualization (equal); formal analysis (equal); writing – review and editing (equal). **Sarah Worth:** Conceptualization (equal); formal analysis (equal); writing – review and editing (equal). **Archibong Yellow‐Duke:** Conceptualization (equal); formal analysis (equal); writing – review and editing (equal). **Wei‐Han Cheng:** Conceptualization (equal); formal analysis (equal); writing – review and editing (equal). **Michael E. Werner:** Conceptualization (equal); formal analysis (equal); writing – review and editing (equal). **Jonathan Abbas:** Conceptualization (equal); formal analysis (equal); writing – review and editing (equal). **William Donnellan:** Conceptualization (equal); formal analysis (equal); writing – review and editing (equal).

## FUNDING INFORMATION

F. Hoffmann‐La Roche Ltd. provided financial support for this manuscript.

## CONFLICT OF INTEREST STATEMENT

Pankit Vachhani: consultancy: Blueprint Medicines, Incyte, AbbVie, CTI BioPharma Corp, Novartis, Amgen, Pfizer, Genentech, Stemline; speaker's bureau: Incyte; Esprit Ma: employment and equity: Genentech; Tao Xu: employment and stockholder: Roche; Melissa Montez: employment: Genentech; stockholder: Roche; Sarah Worth: employment: University of Alabama at Birmingham; honoraria: JADPRO Expert Conversations in CML, July 2021; membership on an entity's Board of Directors or advisory committees: Blueprint Medicines Advisory Committee, August 2021, and CTI BioPharma Corp Advisory Committee, February 2022; Archibong Yellow‐Duke: employment: Genentech; equity: Genentech, Roche; Wei‐Han Cheng: employment and stock options: AbbVie; Michael E Werner: employment and stockholder: AbbVie; Jonathan Abbas: employment: Tennessee Oncology; consultancy: AbbVie, BMS; speaker's bureau: AbbVie, BMS, Incyte, Morphosys; William Donnellan: consultancy: Janssen, Merck, Pfizer, Amgen, Gilead; research funding: Merck, Pfizer, Amgen.

## ETHICS STATEMENT

Institutional review board approval of the study protocol was obtained prior to study conduct. Studies were conducted in accordance with recognized ethical guidelines including the Declaration of Helsinki, the Council for International Organizations of Medical Science (CIOMS), the Belmont Report, and the US Common Rule.

## PATIENT CONSENT STATEMENT

Institutional review board approval included a waiver of informed consent.

## Supporting information


Data S1

## Data Availability

The data that support the findings of this study have been originated by Flatiron Health, Inc. Requests for data sharing by license or by permission for the specific purpose of replicating results in this manuscript can be submitted to dataaccess@flatiron.com.
