# Supplementary material for: Post‐remission cytopenia management in patients with AML treated with venetoclax in combination with hypomethylating agents: Pre‐ versus post‐VIALE‐A real‐world experience from a predominantly US community setting
Source: Cancer Med. 2023 Aug 11;12(17):17914–23. doi: 10.1002/cam4.6430 (PMC10523977; doi:10.1002/cam4.6430)
Supplement: Supplementary file 1 — Data S1 [file CAM4-12-17914-s001.docx]

**Supplementary Information**

**Supplementary Methods**

Treatment schedule modifications included:

1. In-cycle interruptions: dose hold/holds in the middle of a cycle.
2. Cycle delays: dose hold within the last days of a cycle that subsequently delays the start of the next cycle.
3. Dose schedule per cycle changes: where number of dosing days per cycle changes from one cycle to another, e.g., a 28-day dosing cycle converts to a 21-day dosing cycle (although the cycle itself remains the same length [28-days]).

**
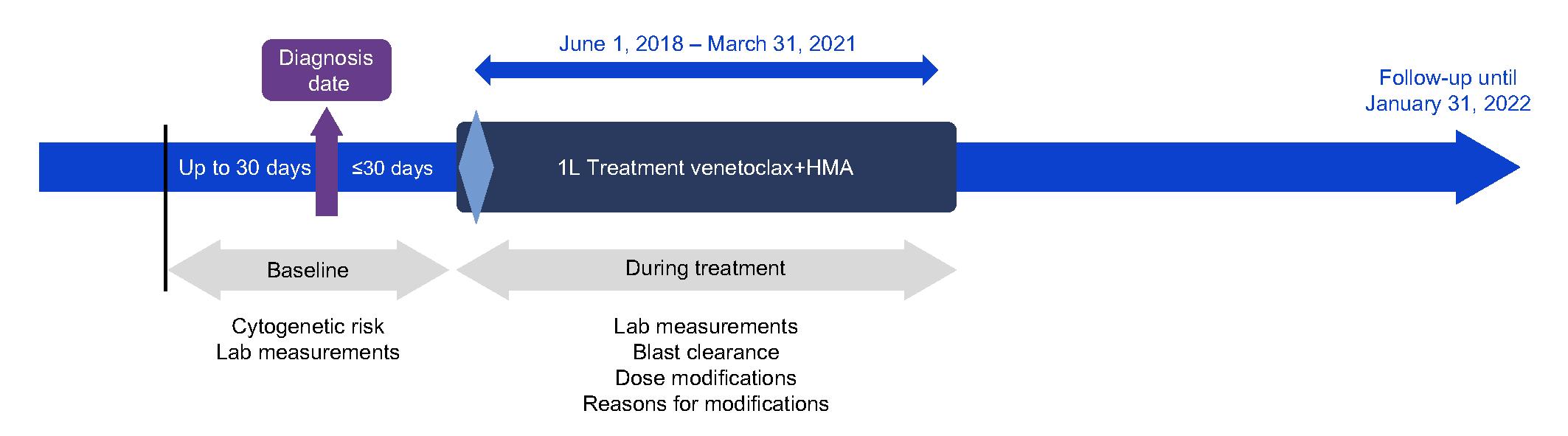
Supplementary Figure 1. Study design**

The light blue diamond denotes index date, where treatment initiation occurred up to 14 days prior to the recorded diagnosis date, this may be considered the start of 1L, this is anticipated to occur for a small proportion of patients; the baseline period remains the same window of up to 30 days prior to the diagnosis date.

1L, first-line; HMA, hypomethylating agents.

**Supplementary Figure 2. Patient attrition**


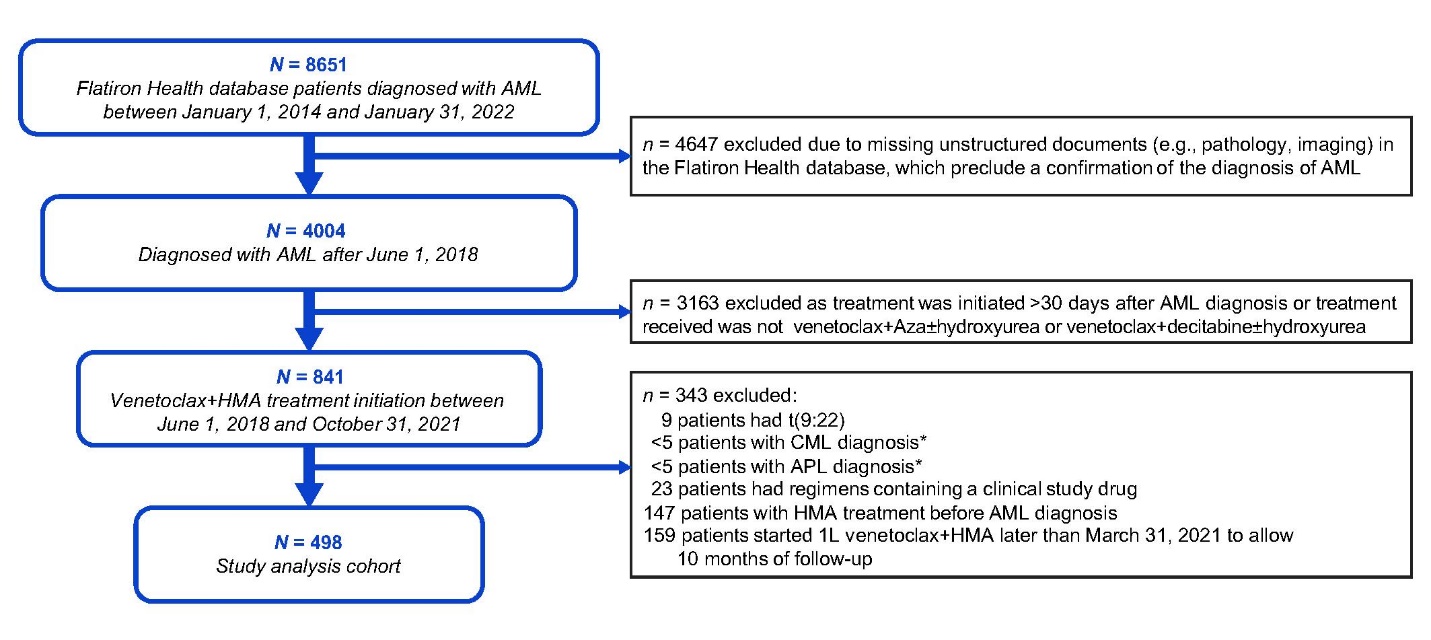


*Per the threshold accepted by the National Center for Health Statistics and the Agency for Healthcare Research and Quality, detailed in the Federal Committee’s Statistical Policy, 2005, any instances where there are fewer than five patients for a particular characteristic or variable have been described as such to eliminate potential patient re-identification.

1L, first-line; AML, acute myeloid leukemia; APL, acute promyelocytic leukemia; Aza, azacitidine; CML, chronic myeloid leukemia; HMA, hypomethylating agents.

**Supplementary Figure 3. Proportion of patients who achieved remission (<5% BM blast) and underwent venetoclax schedule modifications in the pre- versus post-VIALE-A cohorts**


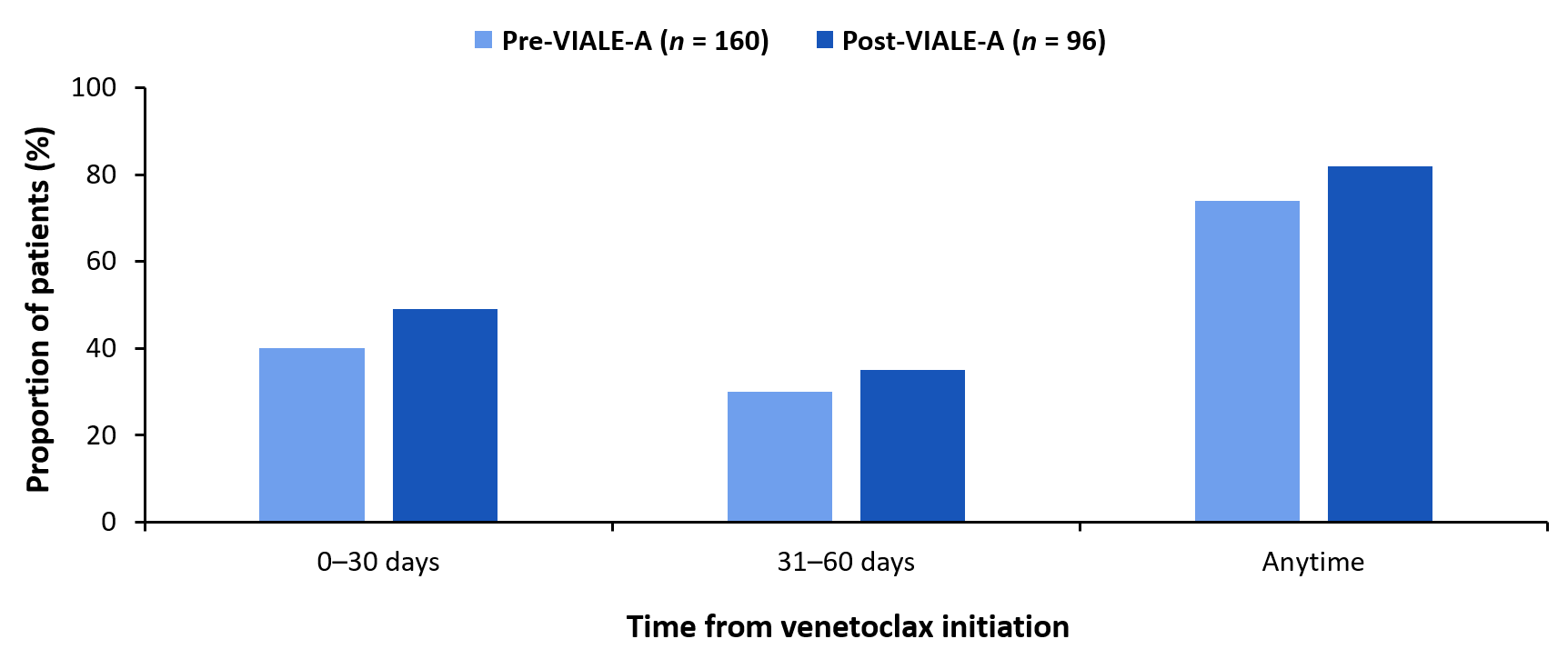


BM, bone marrow.

**Supplementary Figure 4. Time varying survival analysis of patients with newly diagnosed AML receiving venetoclax+HMA who converted from 28-day to 21-day dose schedule cycle versus stayed on 28-day dose schedule cycle after remission for all patients who responded (A), patients who achieved rwCR/CRi (B) and patients who achieved rwCR/CRh (C)**


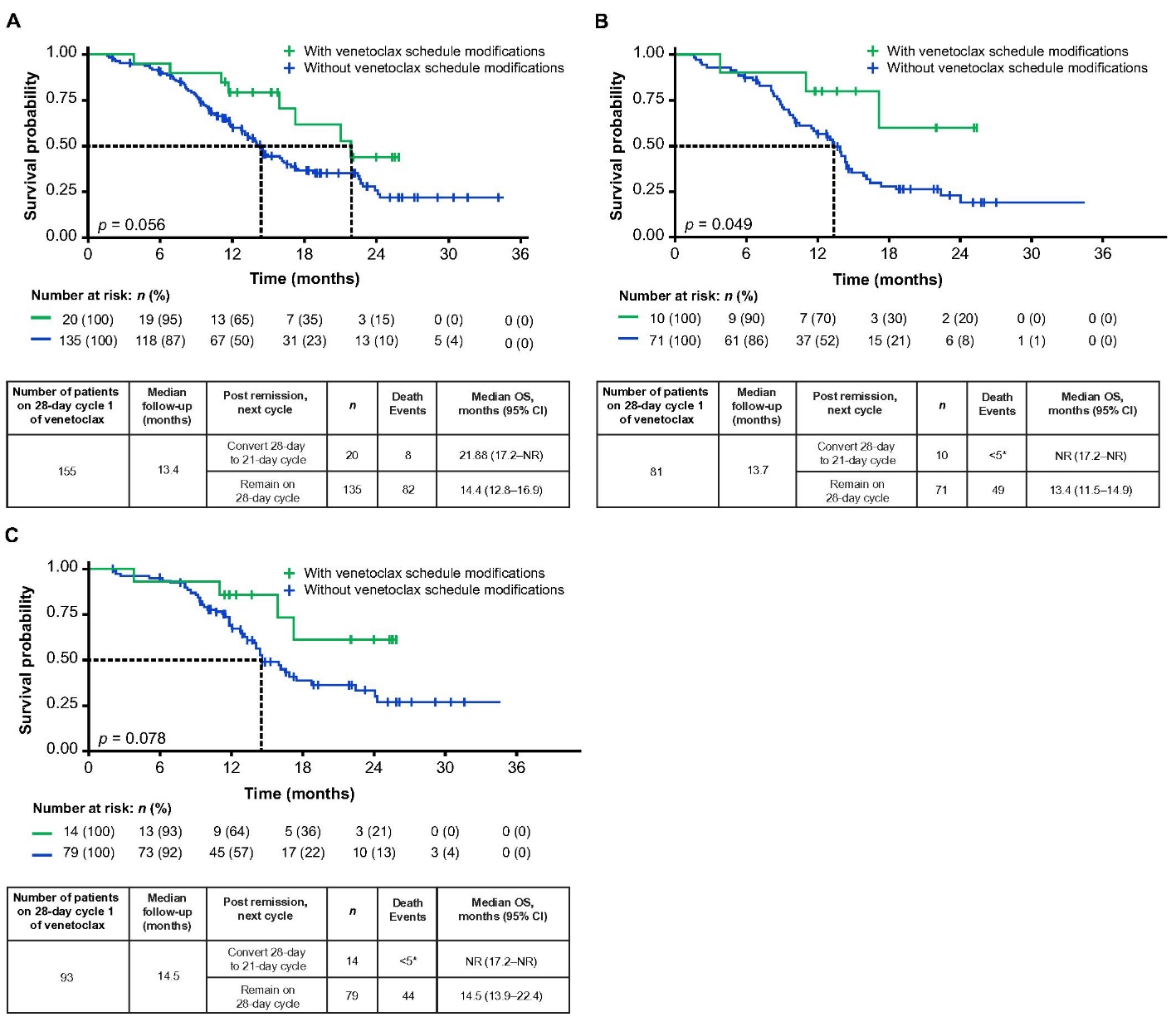


Per the threshold accepted by the National Center for Health Statistics and the Agency for Healthcare Research and Quality, detailed in the Federal Committee’s Statistical Policy, 2005, any instances where there are fewer than five patients for a particular characteristic or variable have been described as such to eliminate potential patient re-identification.
AML, acute myeloid leukemia; CI, confidence interval; CRh, complete response with partial hematologic recovery; CRi, complete response with incomplete hematologic recovery; HMA, hypomethylating agents; NR, not reached; OS, overall survival; rwCR, real-world complete response.

**Supplementary Table 1.** **Patient demographic and characteristics among patients who responded with versus without venetoclax treatment schedule modification**

|  | **Patients with treatment schedule modifications**  **(*n* = 201)** | **Patients without treatment schedule modifications**  **(*n* = 54)** | ***p* value** |
| --- | --- | --- | --- |
| **Age at diagnosis (continuous)** |  |  |  |
| Mean (SD) | 73.7 (7.9) | 73.8 (7.2) | .674 |
| Median (Min, Max) | 75.0 (36.0, 84.0) | 75.5 (53.0, 83.0) |  |
| **Age at diagnosis (categorical), *n* (%)** |  |  |  |
| <18 years | 0 | 0 | .539 |
| 18–64 years | 23 (11.4) | 7 (13.0) |  |
| 65–74 years | 76 (37.8) | 16 (29.6) |  |
| ≥75 years | 102 (50.7) | 31 (57.4) |  |
| **Sex, *n* (%)** |  |  |  |
| Female | 81 (40.3) | 22 (40.7) | 1 |
| Male | 120 (59.7) | 32 (59.3) |  |
| **Year of diagnosis, *n* (%)** |  |  |  |
| 2018 | 14 (7.0) | <5 (<9.3)* | .318 |
| 2019 | 67 (33.3) | 16 (29.6) |  |
| 2020 | 93 (46.3) | 31 (57.4) |  |
| 2021 | 27 (13.4) | <5 (<9.3)* |  |
| **AML type, *n* (%)** |  |  |  |
| *De novo* AML | 146 (72.6) | 37 (68.5) | .67 |
| Secondary AML | 55 (27.4) | 17 (31.5) |  |
| **ELN classification, *n* (%)** |  |  |  |
| Adverse | 80 (39.8) | 27 (50.0) | .57 |
| Intermediate | 51 (25.4) | 12 (22.2) |  |
| Favorable | 29 (14.4) | <5 (<9.3)* |  |
| Inconclusive | 24 (11.9) | 6 (11.1) |  |
| No genomic test | 17 (8.5) | 5 (9.3) |  |
| **Practice type, *n* (%)** |  |  |  |
| Academic | 45 (22.4) | 13 (24.1) | .937 |
| Community | 156 (77.6) | 41 (75.9) |  |

*Per the threshold accepted by the National Center for Health Statistics and the Agency for Healthcare Research and Quality, detailed in the Federal Committee’s Statistical Policy, 2005, any instances where there are fewer than five patients for a particular characteristic or variable have been described as such to eliminate potential patient re-identification.

AML, acute myeloid leukemia; ELN, European Leukemia Net; Max, maximum; Min, minimum; SD, standard deviation.

**Supplementary Table 2.** **Patient demographic and characteristics amongst patients who achieved rwCR/CRi with or without venetoclax treatment schedule modification**

|  | **Patients with treatment schedule modifications**  **(*n* = 112)** | **Patients without treatment schedule modifications**  **(*n* = 25)** | ***p* value** | |
| --- | --- | --- | --- | --- |
| **Age at diagnosis (continuous)** |  |  |  | |
| Mean (SD) | 73.4 (8.1) | 75.6 (5.0) | .09 | |
| Median (Min, Max) | 75.0 (36.0, 83.0) | 76.0 (63.0, 83.0) |  | |
| **Age at diagnosis (categorical), *n* (%)** |  |  |  | |
| <18 years | 0 | 0 | .349 | |
| 18–64 years | 13 (11.6) | <5 (<20.0)* |  | |
| 65–74 years | 40 (35.7) | 7 (28.0) |  | |
| ≥75 years | 59 (52.7) | 17 (68.0) |  | |
| **Sex, *n* (%)** |  |  |  | |
| Female | 40 (35.7) | 15 (60.0) | .044 | |
| Male | 72 (64.3) | 10 (40.0) |  | |
| **Year of diagnosis, *n* (%)** |  |  |  | |
| 2018 | 11 (9.8) | <5 (<20.0)* | .19 | |
| 2019 | 36 (32.1) | 10 (40.0) |  | |
| 2020 | 51 (45.5) | 14 (56.0) |  | |
| 2021 | 14 (12.5) | 0 |  | |
| **AML type, *n* (%)** |  |  |  | |
| *De novo* AML | 76 (67.9) | 15 (60.0) | .604 | |
| Secondary AML | 36 (32.1) | 10 (40.0) |  | |
| **ELN classification, *n* (%)** |  |  |  | |
| Adverse | 47 (42.0) | 12 (48.0) | .74 |  |
| Intermediate | 27 (24.1) | 8 (32.0) |  |  |
| Favorable | 16 (14.3) | <5 (<20.0)* |  |  |
| Inconclusive | 13 (11.6) | <5 (<20.0)* |  |  |
| No Genomic Test | 9 (8.0) | <5 (<20.0)* |  |  |
| **Practice type, *n* (%)** |  |  |  |  |
| Academic | 28 (25.0) | 7 (28.0) | .954 |  |
| Community | 84 (75.0) | 18 (72.0) |  |  |

*Per the threshold accepted by the National Center for Health Statistics and the Agency for Healthcare Research and Quality, detailed in the Federal Committee’s Statistical Policy, 2005, any instances where there are fewer than five patients for a particular characteristic or variable have been described as such to eliminate potential patient re-identification.

AML, acute myeloid leukemia; ELN, European Leukemia Net; Max, maximum; Min, minimum; rwCR/CRi, real-world complete response/complete response with incomplete hematologic recovery; SD, standard deviation.

**Supplementary Table 3. Patient demographic and characteristics amongst patients who achieved rwCR/CRh with or without venetoclax treatment schedule modification**

|  | **Patients with treatment schedule modifications**  **(*n* = 130)** | **Patients without treatment schedule modifications**  **(*n* = 29)** | ***p* value** |
| --- | --- | --- | --- |
| **Age at diagnosis (continuous)** |  |  |  |
| Mean (SD) | 72.6 (8.5) | 74.4 (7.2) | .251 |
| Median (Min, Max) | 74.0 (36.0, 84.0) | 76.0 (54.0, 83.0) |  |
| **Age at diagnosis (categorical), *n* (%)** |  |  |  |
| <18 years | 0 | 0 | .687 |
| 18–64 years | 19 (14.6) | <5 (<17.2)* |  |
| 65–74 years | 47 (36.2) | 9 (31.0) |  |
| ≥75 years | 64 (49.2) | 17 (58.6) |  |
| **Sex, *n* (%)** |  |  |  |
| Female | 53 (40.8) | 11 (37.9) | .942 |
| Male | 77 (59.2) | 18 (62.1) |  |
| **Year of diagnosis, *n* (%)** |  |  |  |
| 2018 | 11 (8.5) | <5 (<17.2)* | .221 |
| 2019 | 45 (34.6) | 8 (27.6) |  |
| 2020 | 56 (43.1) | 18 (62.1) |  |
| 2021 | 18 (13.8) | <5 (<17.2)* |  |
| **AML type, *n* (%)** |  |  |  |
| *De novo* AML | 97 (74.6) | 21 (72.4) | .992 |
| Secondary AML | 33 (25.4) | 8 (27.6) |  |
| **ELN classification, *n* (%)** |  |  |  |
| Adverse | 49 (37.7) | 16 (55.2) | .227 |
| Intermediate | 35 (26.9) | 6 (20.7) |  |
| Favorable | 19 (14.6) | <5 (<17.2)* |  |
| Inconclusive | 16 (12.3) | <5 (<17.2)* |  |
| No genomic test | 11 (8.5) | <5 (<17.2)* |  |
| **Practice type, *n* (%)** |  |  |  |
| Academic | 28 (21.5) | 7 (24.1) | .954 |
| Community | 102 (78.5) | 22 (75.9) |  |

*Per the threshold accepted by the National Center for Health Statistics and the Agency for Healthcare Research and Quality, detailed in the Federal Committee’s Statistical Policy, 2005, any instances where there are fewer than five patients for a particular characteristic or variable have been described as such to eliminate potential patient re-identification.

AML, acute myeloid leukemia; ELN, European Leukemia Net; Max, maximum; Min, minimum; rwCR/CRh, real-world complete response/complete response with partial hematologic recovery; SD, standard deviation.
